# Fatigue in primary Sjögren’s syndrome (pSS) is associated with lower levels of proinflammatory cytokines: a validation study

**DOI:** 10.1007/s00296-019-04354-0

**Published:** 2019-06-27

**Authors:** Kristen Davies, Kamran Mirza, Jessica Tarn, Nadia Howard-Tripp, Simon J. Bowman, Dennis Lendrem, UK Primary Sjögren’s Syndrome Registry, Frances Hall, Elalaine C. Bacabac, Helen Frankland, Robert Moots, Kuntal Chadravarty, Shamin Lamabadusuriya, Michele Bombardieri, Constantino Pitzalis, Nurhan Sutcliffe, Celia Breston, Nagui Gendi, Karen Culfear, Claire Riddell, John Hamburger, Andrea Richards, Saaeha Rauz, Sue Brailsford, Joanne Dasgin, Joanne Logan, Diarmuid Mulherin, Jacqueline Andrews, Paul Emery, Alison McManus, Colin Pease, David Pickles, Alison Booth, Marian Regan, Jon King, Amanda Holt, Theodoros Dimitroulas, Lucy Kadiki, Daljit Kaur, George Kitas, Abdul Khan, Tracey Cosier, Panthakalam, Kelly Mintrim, Mark Lloyd, Lisa Moore, Esther Gordon, Cathy Lawson, Monica Gupta, John Hunter, Lesley Stirton, Gill Ortiz, Elizabeth Price, Suzannah Pelger, Claire Gorman, Balinder Hans, Gavin Clunie, Suzanne Lane, Ginny Rose, Sue Cuckow, Michael Batley, Ruby Einosas, Susan Knight, Deborah Symmons, Beverley Jones, Andrew Carr, Suzanne Edgar, Francisco Figuereido, Heather Foggo, Dennis Lendrem, Iain Macleod, Sheryl Mitchell, Christine Downie, Jessica Tarn, James Locke, Shereen Al-Ali, Sarah Legg, Kamran Mirza, Ben Hargreaves, Laura Hetherington, Adrian Jones, Peter Lanyon, Alice Muir, Paula White, Steven Young-Min, Susan Pugmire, Saravanan Vadivelu, Annie Cooper, Marianne Watkins, Anne Field, Stephen Kaye, Devesh Mewar, Patricia Medcalf, Pamela Tomlinson, Debbie Whiteside, Neil McHugh, John Pauling, Julie James, Andrea Dowden, Mohammed Akil, Jayne McDermott, Olivia Godia, David Coady, Elizabeth Kidd, Lynne Palmer, Charles Li, Sarah Bartrum, Dee Mead, Bhaskar Dasgupta, Victoria Katsande, Pamela Long, Olivia Godia, Erin Vermaak, Janet Turner, Usha Chandra, Kirsten MacKay, Stefano Fedele, Ada Ferenkeh-Koroma, Ian Giles, David Isenberg, Helena MaConnell, Nyarko Ahwireng, Stephen Porter, Paul Allcoat, John McLaren, Wan-Fai Ng

**Affiliations:** 1 Musculoskeletal Research Group, Institute of Cellular Medicine, Newcastle University, Newcastle upon Tyne, NE2 4HH UK; 2 Newcastle-upon-Tyne Hospitals NHS Foundation Trust, Newcastle upon Tyne, UK; 3 University Hospitals Birmingham NHS Foundation Trust, Birmingham, UK; 4 National Institute of Health Research Biomedical Research Centre, Newcastle University, Newcastle upon Tyne, UK

**Keywords:** Fatigue, Primary Sjögren’s syndrome, Proinflammatory, Cytokines

## Abstract

Primary Sjögren’s syndrome (pSS) is a chronic autoimmune rheumatic disease with symptoms including dryness, fatigue, and pain. The previous work by our group has suggested that certain proinflammatory cytokines are inversely related to patient-reported levels of fatigue. To date, these findings have not been validated. This study aims to validate this observation. Blood levels of seven cytokines were measured in 120 patients with pSS from the United Kingdom Primary Sjögren’s Syndrome Registry and 30 age-matched healthy non-fatigued controls. Patient-reported scores for fatigue were classified according to severity and compared to cytokine levels using analysis of variance. The differences between cytokines in cases and controls were evaluated using Wilcoxon test. A logistic regression model was used to determine the most important identifiers of fatigue. Five cytokines, interferon-γ-induced protein-10 (IP-10), tumour necrosis factor-α (TNFα), interferon-α (IFNα), interferon-γ (IFN-γ), and lymphotoxin-α (LT-α) were significantly higher in patients with pSS (*n* = 120) compared to non-fatigued controls (*n* = 30). Levels of two proinflammatory cytokines, TNF-α (*p* = 0.021) and LT-α (*p* = 0.043), were inversely related to patient-reported levels of fatigue. Cytokine levels, disease-specific and clinical parameters as well as pain, anxiety, and depression were used as predictors in our validation model. The model correctly identifies fatigue levels with 85% accuracy. Consistent with the original study, pain, depression, and proinflammatory cytokines appear to be the most powerful predictors of fatigue in pSS. TNF-α and LT-α have an inverse relationship with fatigue severity in pSS challenging the notion that proinflammatory cytokines directly mediate fatigue in chronic immunological conditions.

**Electronic supplementary material:**

The online version of this article (10.1007/s00296-019-04354-0) contains supplementary material, which is available to authorized users.

## Introduction

Fatigue is a complex and disabling symptom affecting between 22 and 30% of the general population resulting in reduced quality of life and associated with substantial economic cost [1–5]. It is a prominent feature of numerous chronic diseases, particularly rheumatic diseases such as primary Sjögren’s syndrome (pSS) [5, 6].

The aetiology of fatigue is complex and likely multifactorial, with both biological and psychosocial elements contributing towards the perception of fatigue. Regarding the biological basis of fatigue, proinflammatory cytokines may play a central role [7]. Fatigue is frequently observed in immune-mediated inflammatory conditions and following infections, as part of a constellation of symptoms termed ‘sickness behaviour’. An adaptive response to infection, sickness behaviour minimises energy expenditure when an organism is in a weakened state following an infection and resolves with the resolution of inflammation [7].

Research previously performed by our group investigated the relationship between proinflammatory cytokines and fatigue in pSS [8]. Howard-Tripp et al. found 14 cytokines to be significantly higher in patients with pSS compared to non-fatigued controls. When patients with pSS were grouped according to fatigue severity, an unexpected inverse relationship was found between fatigue scores and four proinflammatory cytokines, IP-10, TNF-α, LT-α, and IFN-γ. Considering the role cytokines play in the development of the initial inflammatory response, our group postulated that a potentially maladaptive immune response may contribute to the maintenance of persistent fatigue in a chronic inflammatory state as observed in conditions such as pSS. A logistic regression model was created using the cytokines and clinical and disease-specific parameters, which was able to predict fatigue levels with reasonable accuracy (67%). Similar accuracy was reported using only IP-10 and IFN-γ, along with depression and pain scores.

This study aims to validate the previously reported paradoxical observation of an inverse association between proinflammatory cytokines and patient-reported fatigue scores. We measured the serum levels of seven proinflammatory cytokines—interferon-γ-induced protein 10 (IP-10), tumour necrosis factor-α (TNF-α), lymphotoxin-α (LT-α), interferon-γ (IFN-γ), interferon-α (IFN-α), interleukin-12p70 (IL-12p70), and interleukin-17 (IL-17). Six of these were reported to be significantly higher in pSS patients [8] including four demonstrating an inverse relationship with fatigue levels. In addition, while IFN-α was not found to be significantly higher in pSS patients, it has been well documented as a potential inducer of fatigue [9].

## Methods

### Experimental design

The objective of this study was to analyse proinflammatory cytokine and fatigue levels in patients with pSS to validate the previously observed relationship between cytokines and fatigue in pSS. Similar to the original study, we used clinical and biological data to ascertain the most important predictors of fatigue within this patient group.

### Study population

An independent cohort of patients were selected from the United Kingdom Primary Sjögren’s Syndrome Registry (UKPSSR) [10]. The UKPSSR holds clinical, haematological, and demographic data on over 900 patients with pSS and 350 healthy controls across 37 centres in the UK. All patients on UKPSSR fulfil the American European Consensus Group criteria for classification of pSS. This study randomly selected 30 blood samples each from female patients with pSS with either minimal, mild, moderate, or severe fatigue, using a random number generator, for a total of 120 patients. Thirty non-fatigued healthy controls from the UKPSSR were also selected. The North West Research Ethics Committee granted the ethical approval for this study.

### Clinical parameters

The following clinical parameters were collected contemporaneously as the biological samples for this study. Fatigue severity was measured using the Profile of Fatigue (PROF) Questionnaire which has been validated for pSS [11]. Physical fatigue was scored on a scale of 0–7 to group patients into minimal (0–1), mild (2–3), moderate (4–5), and severe (6–7) fatigue groups. Controls were screened for the presence of fatigue using a self-reported questionnaire. None of the controls reported the presence of fatigue or autoimmune disease. Anxiety and depression were measured using the Hospital Anxiety and Depression Score [12].

Disease-specific measures such as the EULAR Sjögren’s Syndrome Disease Activity Index (ESSDAI) and the EULAR Sjögren’s Syndrome Patient-Reported Index (ESSPRI), Schirmer’s test, unstimulated oral salivary flow, and the EULAR Sicca Score were also included [13, 14].

Clinical laboratory data including white cell count (WCC), lymphocytes, neutrophils, haemoglobin, erythrocyte sedimentation rate (ESR), and C-reactive protein (CRP) were measured by the NHS laboratory of the recruiting centre within a day of sample collection.

### Cytokine measurement

Proinflammatory cytokines were measured using biobanked serum samples from the UKPSSR. Seven cytokines were tested: Interferon-γ-induced protein 10 (IP-10), tumour necrosis factor-α (TNF-α), lymphotoxin-α (LT-α), interferon-γ (IFN-γ), interferon-α (IFN-α), interleukin-12p70 (IL-12p70), and interleukin-17 (IL-17). Six cytokines were analysed with cytometric bead array and IL-17 was measured using enzyme-linked immunosorbent assay as previously described [9].

### Statistical analysis

Patient demographic data and clinical data are presented using median and IQR. Statistical significance was determined using Kruskal–Wallis test. Cytokine levels were log transformed prior to analysis of variance, which was used to examine the relationship between the cytokine level and the corresponding fatigue score.

Ordinal logistic regression analysis was used to model fatigue levels using all cytokines, WCC, lymphocytes, neutrophils, ESR, CRP, ESSDAI, and dryness scores, as well as depression, pain, and anxiety scores. The tests performed on this data set were robust to FDR multiple test correction.

All statistical analyses and graphical visualizations were performed using R version 3.1.1 and SAS JMP Pro (Version 14) Statistical Data Visualisation software [15, 16].

## Results

### Study population

Patients with pSS were stratified into four groups according to their fatigue levels as previously described [9]. All patients were female and predominantly Caucasian. When comparing fatigue groups, there were statistically significant differences between ESSDAI, ESSPRI, EULAR-SS, WCC, lymphocytes, and BMI (Table 1). Age, disease, and symptom duration were not significantly different between groups. Anti-Ro/La positivity and percentages of each group taking immune-altering medications did not differ significantly between groups.

**Table 1:** Clinical summary for the four separate pSS fatigue groups demonstrating mean and standard deviation for key demographics, haematological, and clinical variables

	Minimal	Mild	Moderate	Severe	*p* value
Age (years)	53 ± 13	58 ± 14	60 ± 12	56 ± 12	Ns
Disease duration (years)	4.4 ± 5.5	7.0 ± 7.7	4.3 ± 4.9	5.1 ± 7.3	Ns
Symptom duration (years)	9 ± 7	12 ± 9	9 ± 7	9 ± 8	Ns
BMI (kg/m^2^)	26 ± 5	24 ± 4	26 ± 6	30 ± 7	**0.0017**
% not taking immune-altering medications	43	23	30	20	Ns
% on hydroxychloroquine	33	43	40	46	Ns
% on prednisolone	7	10	7	20	Ns
% on ‘other’ immune-altering medications	13	24	33	14	Ns
ESSDAI	2.8 ± 2.9	3.3 ± 3.4	3.4 ± 3.7	4.8 ± 3.4	Ns
ESSPRI	2.6 ± 1.6	4.6 ± 1.3	5.8 ± 1.6	8.3 ± 1.0	**< 0.0001**
ESSPRI pain	0.8 ± 0.8	3.4 ± 0.8	5.0 ± 0.8	6.5 ± 0.5	**< 0.0001**
ESSPRI dryness	1.3 ± 1.2	3.5 ± 0.9	4.9 ± 0.8	6.3 ± 0.7	**< 0.0001**
EULAR-SS	3.8 ± 2.5	5.4 ± 2.3	5.9 ± 2.1	8.1 ± 1.6	**< 0.0001**
HADS anxiety (0–21)	4.4 ± 3.2	8.3 ± 3.9	8.9 ± 4.3	10.5 ± 5.2	**< 0.0001**
HADS depression (0–21)	2.4 ± 2.5	4.9 ± 3.1	6.6 ± 3.2	10.6 ± 4.2	**< 0.0001**
Ro+/La+	15	22	14	17	Ns
Ro+/La−	8	5	6	7	Ns
Ro−/La+	1	1	0	0	Ns
Ro−/La−	4	1	9	6	Ns
Hb (g/dL)	13 ± 0.9	13 ± 0.9	13 ± 1.3	13 ± 1.2	Ns
WCC (× 10^9^/L)	5.0 ± 1.3	5.0 ± 1.2	5.6 ± 2.0	6.6 ± 2.6	**0.0042**
Neutrophil (× 10^9^/L)	3.0 ± 1.0	3.1 ± 0.9	3.3 ± 1.3	3.7 ± 1.6	Ns
Lymphocyte (× 10^9^/L)	1.4 ± 0.6	1.3 ± 0.4	1.6 ± 0.6	1.8 ± 0.6	**0.0095**
ESR (mm/h)	27 ± 21	22 ± 18	23 ± 20	24 ± 23	Ns
CRP (mg/L)	1.7 ± 5.2	1.8 ± 5.8	2.2 ± 3.0	4.1 ± 5.6	Ns
IgG (mg/dL)	18 ± 8	16 ± 9	15 ± 7	14 ± 5	Ns

Bold *p* values indicate statistical significance (< 0.05)

### Cytokine differences between patients with pSS and healthy controls

**Fig. 1:**
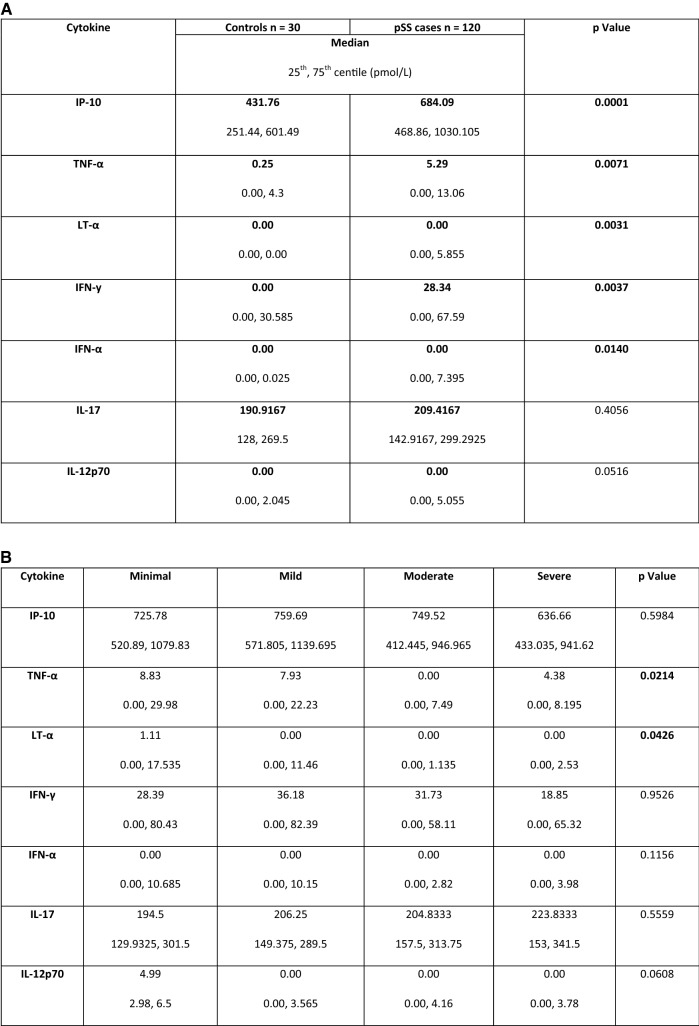
**a** Table of proinflammatory cytokine levels in patients with pSS. Values in the table represent median and 25th, 75th centile (pmol/L). Bold typeface indicates statistical significance of cytokine serum level between fatigue groups as determined by ANOVA. **b** Proinflammatory cytokine levels in patients with pSS. Values in the table represent median and 25th, 75th centile (pmol/L). Bold typeface indicates statistical significance of cytokine serum level between fatigue groups as determined by ANOVA

All proinflammatory cytokines were higher in the pSS population than controls: five proinflammatory cytokines, IP-10, TNFα, LTα, IFN**-**y, and IFNα, were significantly higher in patients with pSS compared to controls, and IL-12p70 was also close to statistical significance (see Fig. 1).

### Cytokine and fatigues scores in pSS

**Fig. 2:**
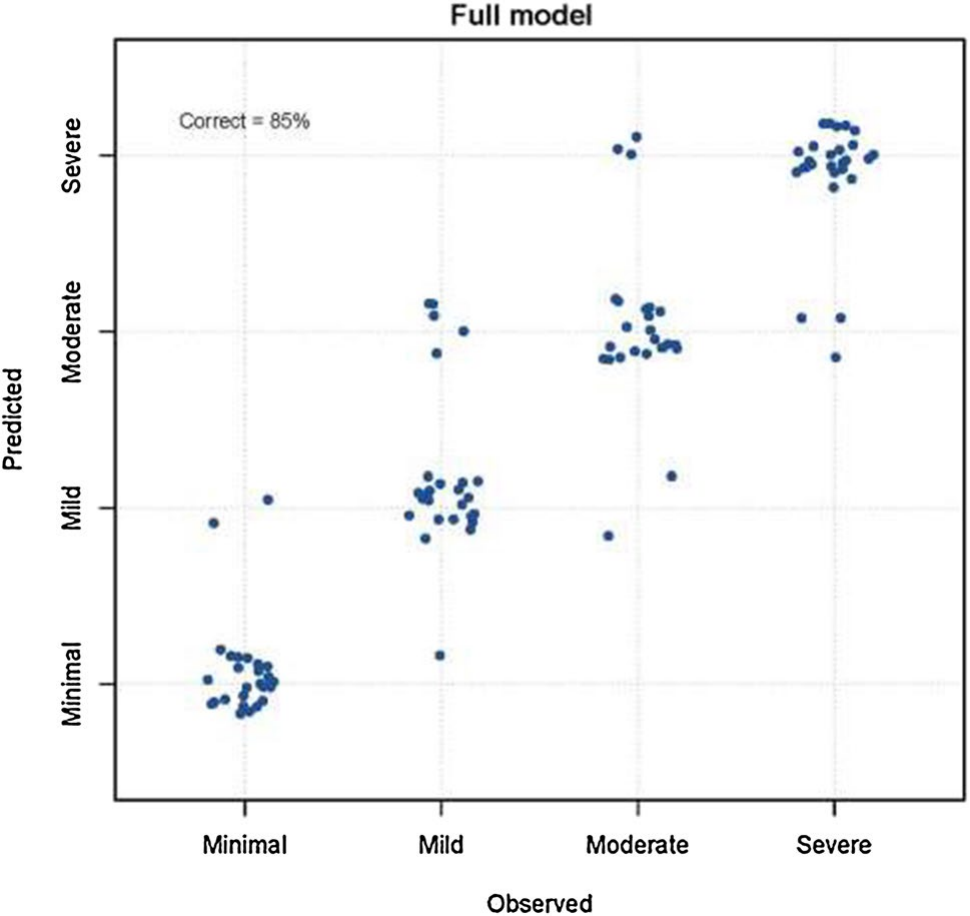
Observed and predicted fatigue levels for the ordinal logistic regression model with all seven cytokines, WCC, lymphocytes, neutrophils, ESR, CRP, ESSDAI scores, dryness scores, depression, pain, and anxiety. This full model predicts fatigue level correctly in 85% of cases

All four of the cytokines evaluated in the original study—IP-10, IFN-y, TNF-α, and LT-α—showed an inverse relationship with patient-reported levels of fatigue. This was statistically significant for TNF-α (*p* = 0.021) and LT-α (*p* = 0.043). Overall, the previously reported trend of higher cytokine levels associated with reduced fatigue scores in pSS patients was replicated—see Fig. 2.

### Proinflammatory cytokines and fatigue severity in pSS

Given the replication of an inverse relationship between proinflammatory cytokines and fatigue in pSS patients, we ran an additional analysis as in the original paper [8]. This logistic regression model was originally used to establish whether fatigue levels could be identified using cytokines, disease-specific and clinical parameters as well as pain, anxiety, and depression. We repeated this analysis by initially examining whether cytokines and/or covariates could successfully identify fatigue category. We then looked at the effect of each cytokine on the model. The full model, including all seven cytokines, was able to correctly identify fatigue category in 85% of cases (Fig. 2). This model with all parameters was sensitive to the presence or absence of cytokines, depression, anxiety, and pain, but was robust to the presence or absence of other markers of disease activity (WCC, neutrophils, ESR, CRP, ESSDAI, and dryness). We ran a reduced model including the covariates depression, anxiety, and pain alongside LT-α and TNF-α. The reduced model correctly identified fatigue category in 85% of cases, comparable to the full model. The cytokines IP-10, LT-α, IFN-γ, and IL-17 demonstrated the same inverse relationship with fatigue category.

## Discussion

Our study confirms that pSS patients with higher levels of fatigue had lower levels of the proinflammatory cytokines than patients with pSS with lower levels of fatigue. This inverse relationship was observed for all four of the original cytokines, though it was statistically significant only for LT-α and TNF-α.

LT-α and TNF-α are closely related cytokines and are both produced by an activated Th1 response. Howard-Tripp et al. discussed the possibility that dysregulation of Th1 responses could be affiliated with the development of fatigue with the potential that Th2 responses could be important in maintaining fatigue, as demonstrated in CFS [17]. The cytokine abnormalities in CFS, however, have been inconsistent, possibly complicated by confounding psychosocial factors.

There is a documented association between Type 1 IFN signature, often implicated in the pathogenesis of pSS, and TNF-α in autoimmune disease [18]. Data from our research group, however, has recently demonstrated no association with IFN-α or Type I IFN signature with fatigue in pSS [19]. Equally, our findings of the relationship between TNF-α and fatigue would explain the failure in fatigue reduction in patients treated with anti-TNF-α medications [20].

Taken together, our data from this and previous study do not support the simple concept of higher levels of inflammation leading to worse fatigue. Instead, we propose that regulatory mechanisms of inflammation may be responsible for the maintenance of fatigue after the initial proinflammatory response. The presence of persistent immune challenge results in chronic inflammation, which may triggers an inappropriate or exaggerated anti-inflammatory response. Such anti-inflammatory mechanisms may in turn have a role in the persistence of fatigue. In other words, we postulated that the exaggerated or inappropriate immune regulation turns what was an adaptive behavioural response, sickness behaviour, into a persistent, pathological response resulting in chronic fatigue with or without concomitant pain and depression. Identifying such anti-inflammatory mechanisms may give clue to treatment of fatigue in pSS.

The strengths of this validation study include (1) a large, clinically well-defined patient group; (2) minimal demographic variation between fatigue groups in the pSS cohort; (3) the use of validated measures in pSS; and (4) consistency of methods with the original study.

Limitations of this study include the (1) cross-sectional nature of this study; (2) fluctuations of cytokine levels could be influenced by multiple factors; (3) while the original findings have been validated in an independent cohort taken from the UKPSSR, a full external validation in another cohort is needed; and (4) using an all-female cohort, similar to the original study, while minimising the potential gender differences in cytokine profile, means our findings may not be generalisable to men with pSS.

## Conclusion

This study has validated our previous observation of an inverse relationship between proinflammatory cytokines and fatigue levels in pSS. Our data further challenges the notion that proinflammatory cytokines directly mediate fatigue in pSS. Further studies to determine whether the model also applies to other chronic immune-mediated inflammatory conditions and to explore the possible mechanisms of this inverse relationship between proinflammatory cytokines and fatigue levels are warranted.

## Electronic supplementary material

Below is the link to the electronic supplementary material.

Supplementary material 1 (DOCX 6992 kb)
