# Supplementary material for: Fatigue in primary Sjögren’s syndrome (pSS) is associated with lower levels of proinflammatory cytokines: a validation study
Source: Rheumatol Int. 2019 Jun 27;39(11):1867–73. doi: 10.1007/s00296-019-04354-0 (PMC6791914; doi:10.1007/s00296-019-04354-0)
Supplement: Supplementary file 1 — Supplementary material 1 (DOCX 6992 kb) [file 296_2019_4354_MOESM1_ESM.docx]

**Supplementary Appendix**

- 1. **Reduced Model**

**
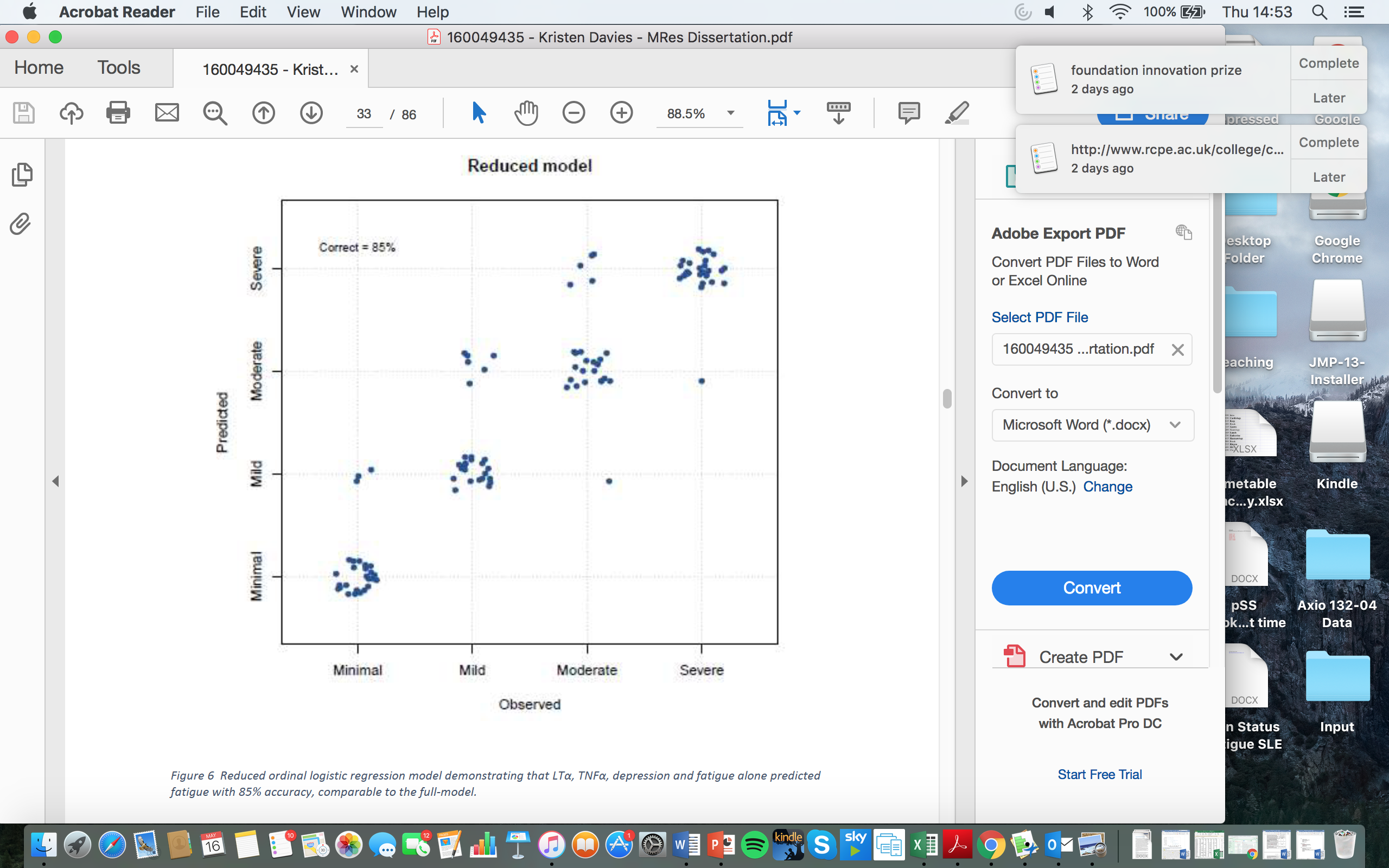
**

### Reduced ordinal logistic regression model involving the parameters of pain, depression, dryness, TNF-α and LT-α.

**1.2 – Confusion Matrix**


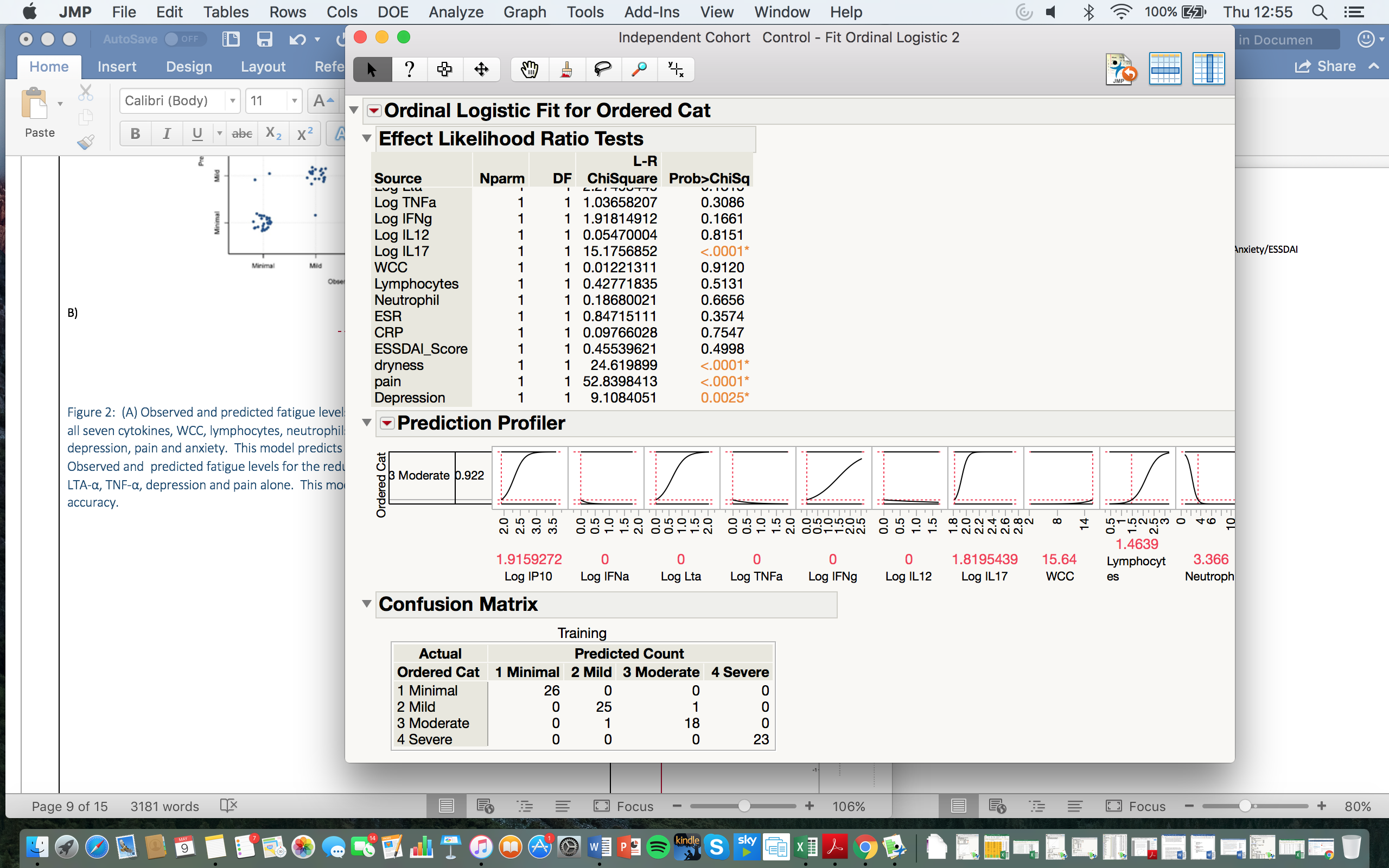


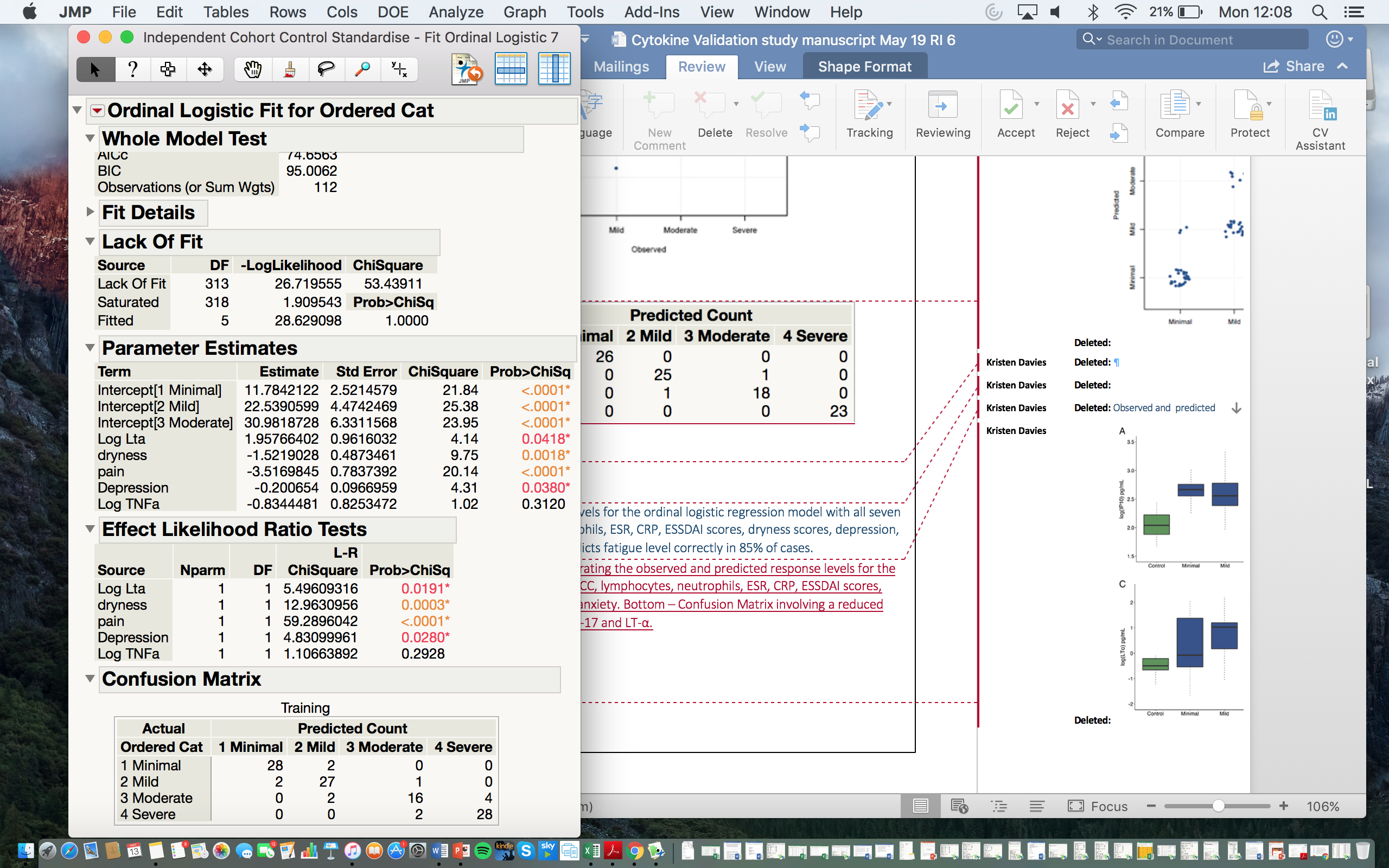


### Top - Confusion Matrix demonstrating the observed and predicted response levels for the full model with all seven cytokines, WCC, lymphocytes, neutrophils, ESR, CRP, ESSDAI scores, dryness scores, depression, pain and anxiety.

### Bottom – Confusion Matrix involving a reduced model of pain, depression, dryness, TNF-α and LT-α. In both cases, most observations are on the diagonal axis indicating good concordance between observed and predicted fatigue levels.

**1.3 ROC Curves for Full and Reduced Models**


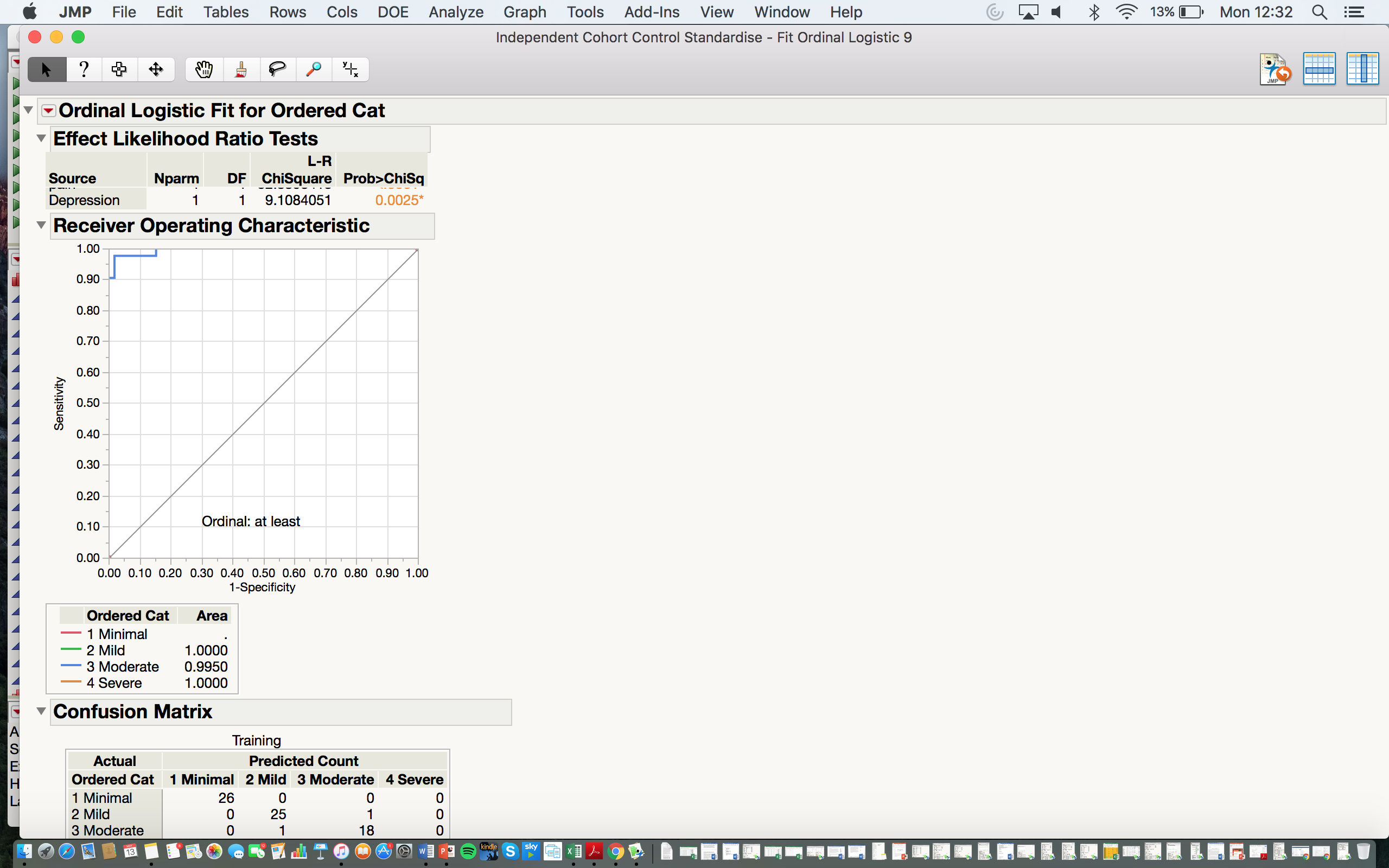


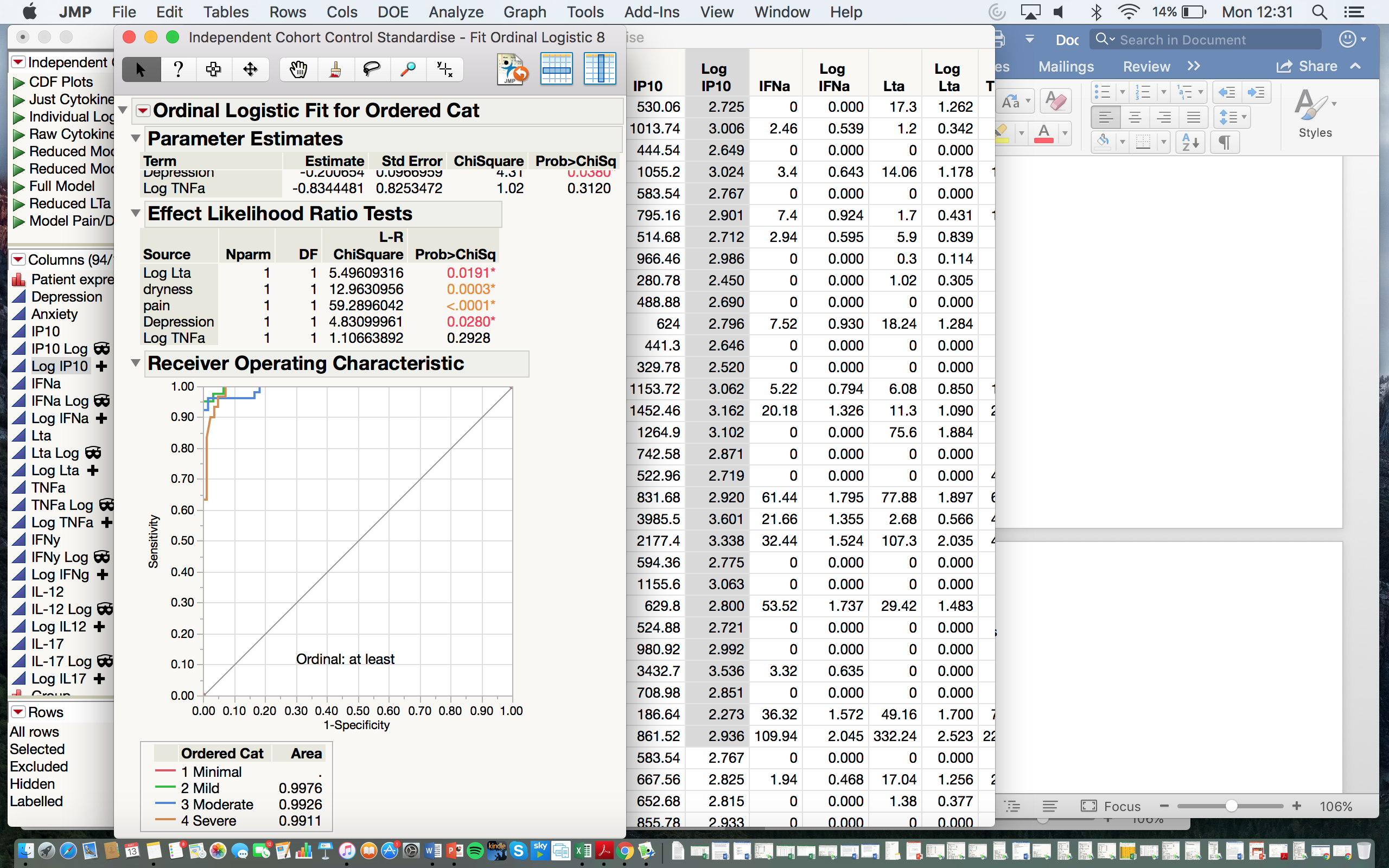


### Top – ROC Curve demonstrating the observed and predicted response levels for the full model with all seven cytokines, WCC, lymphocytes, neutrophils, ESR, CRP, ESSDAI scores, dryness scores, depression, pain and anxiety.

### Bottom – ROC Curve involving a reduced model of pain, depression, dryness, TNF-α and LT-α.
